# Canine Silica Urolithiasis in Mexico (2005–2018)

**DOI:** 10.1155/2020/8883487

**Published:** 2020-10-21

**Authors:** Claudia Iveth Mendoza-López, Javier Del-Angel-Caraza, María Alejandra Aké-Chiñas, Israel Alejandro Quijano-Hernández, Marco Antonio Barbosa-Mireles

**Affiliations:** Hospital Veterinario para Pequeñas Especies de la Facultad de Medicina Veterinaria y Zootecnia, Universidad Autónoma del Estado de México, Jesús Carranza 203 Col. Universidad, Toluca CP 50130, Mexico

## Abstract

A higher frequency of canine silica urolithiasis is found in Mexico, unlike <1–8% in other countries. The causes and risk factors for this pathology are unknown. However, we consider the consumption of high amounts of silica from the solid diet or dissolved in water as the only hypothesis. This study aimed to identify the risk factors for silica urolithiasis in dogs from Mexico. A total of 1383 clinical cases of canine urolithiasis were included in this study; the uroliths were analyzed to determine their mineral composition by stereoscopic microscopy and infrared spectroscopy. Of these cases, 12.94% were considered pure silica uroliths; however, considering the mixed and compound uroliths, the frequency increased to 17.42%. Male dogs aged >6 years and large breeds, especially Labradors and Golden retrievers, were at significant risk for this disease. 98.88 % of the clinical cases studied were found in the central axis of the country, considering this finding as a possible geographical risk factor to be analyzed in another study.

## 1. Introduction

Urolithiasis is a chronic disease present in different dog populations globally. Knowledge of the mineral composition of the uroliths together with the clinical history and other diagnostic tests of the patient allow us to understand the pathophysiological mechanisms and the risk factors that contribute to the formation of uroliths, thus providing the necessary information to design a therapeutic protocol in order to prevent its recurrence in the long term.

According to their mineral composition, there are different types of uroliths: those composed of struvite, calcium oxalate, purines, silica, calcium phosphate, or cystine, and those composed of a mixture of minerals known as mixed and compound uroliths. In the epidemiology of urolithiasis in dogs, it is reported that struvite and calcium oxalate are the most common minerals representing more than 70% of the cases [1–3]. The frequency of other types of uroliths varies considerably depending on the geographical area [4–6]. Specifically, silica urolithiasis has been reported in most studies with a frequency less than 1% [3, 7, 8]; however, three studies reported higher frequencies in the United States 6.7% [9], Switzerland 8% [6], and Mexico 9.2% [4].

This type of urolithiasis has been described in different animal species, such as ruminants, and is linked to the consumption of grasses with high silica content [10, 11]. In humans, it has been associated with the chronic consumption of drugs that contain silicates [12]. Since certain medications are made of aluminum and magnesium silicate, or use colloidal silicon dioxide as a binder, lubricant, absorbent, or stabilizer of emulsions, the chronic intake of these drugs can contribute to the formation of silica stones [13]. Another factor that has been associated with the development of silica urolithiasis is the consumption of water with a high content of silica [14]. In addition, the consumption of clay rich in silica has been described as a food condiment, or for pica [15].

In dogs, the physiopathological mechanism of and risk factors for this type of urolithiasis have not been clearly described. It has been proposed that silica urolithiasis develops due to the consumption of low-quality pet food, which contains vegetable ingredients with high levels of silica added as a cheap source of protein [16], or by the consumption of water with high silica levels, such as the local groundwater [4]. In a study in Kenya, the consumption of corn and/or water with a high concentration of silica was suggested as a possible cause of this disease in native dogs [17]. In epidemiological studies, it has been reported that the majority of canine cases occur in the males of large breeds, such as Golden retrievers, Labradors, and German Shepherds, and the disease rarely occurs in small animals, such as Schnauzers, Lhasa Apsos, and Shih Tzus, or in crossbreed dogs [18, 19].

Our observations in the study of a population of dogs with urolithiasis in different geographical areas of Mexico enabled us to detect a higher frequency of silica urolithiasis (9.23–55%) [4, 20, 21] than that described in other international reports [6, 17]. Therefore, the objective of this study was to perform an epidemiological analysis in the affected population with this pathology and to identify the risk factors involved.

## 2. Materials and Methods

We analyzed data pertinent to cases of urolithiasis in dogs referred to the Uroliths Analysis Laboratory of the Hospital Veterinario para Pequeñas Especies de la Facultad de Medicina Veterinaria y Zootecnia de la Universidad Autónoma del Estado de Mexico (UAL-UAEMex), located in the city of Toluca, Mexico, between 2005 and 2018.

The uroliths analyzed were referred by veterinarians from different states of the country. The samples were accompanied by an input form containing information and the clinical history of the patient. Among the cases detected with silica urolithiasis, the following variables were analyzed: gender, age, breed, breed size, location of the stones within the urinary tract, physical characteristics of the uroliths, and the geographical location of the clinical cases in the country.

The ages were classified into four groups: under the age of 1 year, 1 to 5 years, 6 to 10 years, and older than 10 years. The breed size was classified as small breeds (less than 10 kg) or large breeds (more than 10 kg) [22]. The physical characteristics of the uroliths (shape, size, weight, and hardness, compared to other minerals), as well as the urinary pH measured by dipstick testing at the time of the diagnostic approach, were described. The geographical regions of the cases were also recorded.

### 2.1. Determination of the Mineral Composition of the Uroliths

For the determination of the mineral composition of the uroliths, a physico-chemical analysis was performed. The physical characteristics of the outer surface of the uroliths were evaluated by direct inspection or with stereoscopic microscopy (Stemi DV4 Stereo-Microscope, Zeiss, NY, USA). In order to observe the internal architecture of the uroliths and to differentiate the layers that compose them, it was necessary to cut the uroliths in half. However, this was only done to those with a diameter of >5 mm. The chemical composition of the uroliths was determined by analyzing each of the layers of the uroliths by means of infrared spectroscopy (FT-IR Spectrum two, Perkin Elmer, OH, USA). Uroliths <5 mm were crushed and analyzed as a single layer.

The qualitative interpretation of the spectra obtained was based on what has been described by Hidalgo et al. [23], and for the quantitative analysis of the minerals, struvite, calcium oxalate, calcium phosphate, cystine, purines, and mixtures of these, an electronic library of reference spectra (NICODOM IR Kidney stones1668 spectra, Praha, Czech Republic) was used in addition to a library developed in our laboratory, specifically for silica and its mixtures with other minerals [24]. For the purposes of this study, the term “calcium oxalate” (CaOx) includes the monohydrated and the dehydrated versions, or mixtures of both, and the term “purines” includes ammonium urate and uric acid.

### 2.2. Classification of Uroliths

The uroliths were classified as pure when their structure was formed by a single layer or when the different layers had a similar composition, with more than 70% being composed of a particular mineral (e.g., 80% struvite and 20% CaOx, or 100% struvite); as mixed when the mineral mixture presented a composition with less than 70% of a particular mineral (e.g,. 60% struvite and 40% CaOx); and as compound when the layers of urolith had different mineral compositions as described by Osborne et al. [25], for example, a silica nucleus, a shell of silica stone mixed with struvite, and surface crystals composed of struvite.

### 2.3. Measurement of the Hardness of the Uroliths

The hardness of the uroliths was measured using a portable hardness testing system (Portable durometer THB 100 ERWEKA America Inc, NJ-USA). Each urolith was placed in the durometer, and the force required to cause it to break was recorded, giving the result of the maximum force in kg-force (kgf). This procedure was performed only on five uroliths of different mineral composition (struvite, calcium oxalate, silica, ammonium urate, and cystine) that were complete uroliths, spherical in shape, 5 mm in diameter, and 0.25 g in weight.

### 2.4. Control Group

Comparisons were made between groups to determine the risk factors for the formation of urinary silica stones. In order to do this, two control groups were necessary. The population of the control groups was three times higher than the number of cases of silica urolithiasis in the study. The first control group was obtained at random from the hospital population of new patients that attended a consultation for the first time, using the database of 2016 and 2017 from our veterinary hospital. For this control group, we took into account the data from clinical cases of dogs that presented with any type of disease, excluding those with kidney disease or diseases of the lower urinary tract such as urolithiasis, bacterial urinary tract infections, urinary tract neoplasms, micturition disorders, or prostatic disease. The second control group was obtained at random from the population of urinary stone-forming dogs, using the database of the UAL-UAEMex. We included all types of uroliths, excluding silica uroliths.

### 2.5. Statistical Analysis

The data were captured in a spreadsheet, and descriptive statistics were performed using the GraphPad Prism 6.0 program (https://www.graphpad.com) to determine *Xi*^2^, OR, and its confidence interval of 95%. Differences were considered significant when *p* < 0.05. For the correction of OR, a logistic regression analysis was performed using the Sigma Plot program [http://www.sigmaplot.co.uk/products/sigmaplot/sigmaplot-details.php] by selecting the risk factors that individually had significance (*p* < 0.2).

## 3. Results

### 3.1. Mineral Composition of Uroliths

Of the total uroliths analyzed, 12.9% (179/1383) were composed of silica and were considered pure. However, 42.8% (46/105) of the mixed uroliths contained between 40 and 60% silica in a mixture with other minerals such as struvite, calcium oxalate, cystine, and ammonium urate, and in the case of compound uroliths, 55.1% (16/29) contained a pure silica nucleus, with stones of silica mixed with calcium oxalate or struvite (Table 1). Considering the total population analyzed, 241 cases (17.4%) of uroliths contained silica.

### 3.2. The Geographic Location of the Cases of Silica Urolithiasis in Mexico

**Figure 1:**
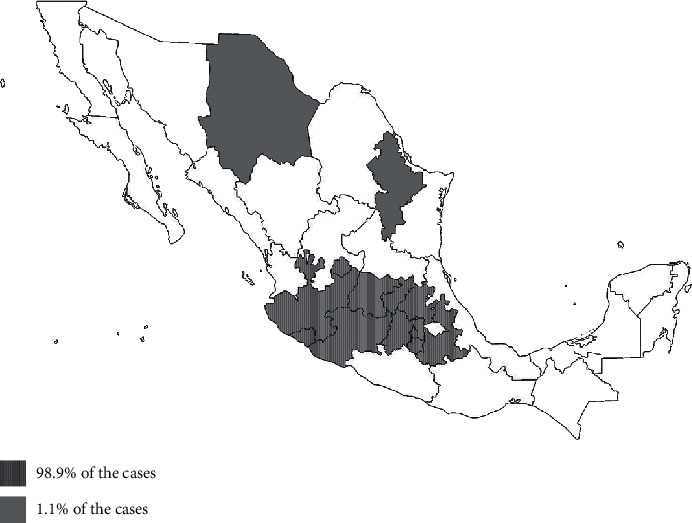
Geographic distribution of cases of silica urolithiasis in Mexico.

When evaluating the cases of silica urolithiasis by region, it is should be noted that 98.9% (*n* = 177) of the cases were located in the central region of the country, in the states of Aguascalientes, Mexico City, Colima, Mexico State, Guanajuato, Hidalgo, Jalisco, Michoacán, Morelos, Nayarit, Puebla, and Queretaro, delimiting a transversal axis; outside this area, the other 1.1% (*n* = 2) was located in the northern area, with a case in Chihuahua and another in Nuevo León, and no cases in the southern region (Figure 1).

In the analysis of animal data with pure silica urolithiasis (*n* = 179), the age range of the dogs was 5 months to 14 years, with a median age of 6 ± 3.1 years. The group of <1 year presented a frequency of 0.5% (*n* = 1), the group 1 to 5 years presented a frequency of 43.6% (*n* = 78), the group 6 to 10 years presented a frequency of 44.1% (*n* = 79), and the group >10 years presented a frequency of 11.7% (*n* = 21). This evidenced a significant risk in animals older than 6 years of age (OR 1.69, CI_0.95_ 1.18–2.40; *p*=0.003) (Table 2).

Assessment of the effect of sex revealed that males were the most affected with 96.6% (*n* = 173) and only 3.3% (*n* = 6) of the cases were affecting females, with a male : female ratio of 28.8 : 1, indicating a significant risk (OR 21.44; CI 0.95, 9.378–49.045; *p* < 0.001).

The studied animals belonged to 33 different breeds; those with the highest frequency were the Labrador 17.9% (*n* = 32), Miniature Schnauzer 11.7% (*n* = 21), Crossbreed 9.5% (*n* = 17), German Shepherd 8.9% (*n* = 16), Golden Retriever 8.9% (*n* = 16), and Poodle 6.7% (*n* = 12), evidencing a significant risk to Labradors and Golden Retrievers (OR 3.00, CI_0.95_ 1.664–5.423; *p* < 0.001), (OR 2.78, CI_0.95_ 1.235–6.278; *p*=0.014), respectively (Table 3).

### 3.3. Breed Size

Of the clinical cases of dogs of a known breed (*n* = 162), 71.6% (*n* = 116) were large breeds and 28.4% (*n* = 46) were small breeds. This indicates a significant risk in large breed of dogs of forming pure silica uroliths (OR 3,542, IC_0.95_ 2.482–5.055, *p*=0.001), and mixed and compound uroliths (OR 3,726, IC_0.95_ 1.730–8.025; *p*=0.001).

### 3.4. Location of the Uroliths in the Urinary Tract

It was possible to obtain information on the location of the uroliths in the urinary tract in 173 cases, the majority of which were found in the lower urinary tract: in the bladder *n* = 90 (52.0%), urethra *n* = 37 (21.4%), and simultaneously in the bladder and urethra *n* = 43 (24.8%). Some uroliths were found in the cranial urinary tract: in the ureter *n* = 1 (0.6%) and the kidney *n* = 1 (0.6%), and some were found in both the cranial and caudal tracts: simultaneously in the kidney and urethra *n* = 1 (0.6%).

### 3.5. Architecture and Hardness of the Silica Uroliths

**Figure 2:**
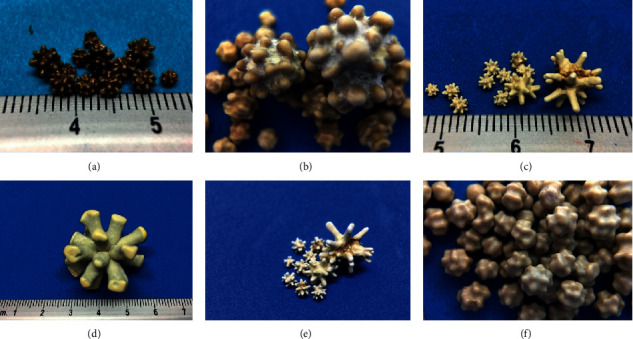
Pure silica uroliths extracted from the lower urinary tract of different dogs. Despite the differences in size, shape, and color of the uroliths, no variations in their chemical composition were found.

The different clinical cases studied had one or more than 30 uroliths, measuring from 1 to 35 mm in diameter, with a weight between 0.01 and 22 g, and presenting in different colors of white, beige, yellow, mustard, coffee, gray, and green. The majority of the uroliths were spherical with radial projections with lengths of 1 to 15 mm, with different thicknesses, and varied in number from 10 to 22 (Jackstone form), and in some cases the tips were blunt and almost imperceptible; and some uroliths were circular or irregular with smooth or rough walls (Figure 2). However, the uroliths with a mixture of silica and other compounds, such as struvite, calcium oxalate, cystine, and ammonium urate, adopted characteristic forms that could be circular, square, irregular, or oval in shape, weighed up to 24 g, and measured up to 40 mm. When analyzing the different layers of the uroliths by electron microscopy, it was observed that in the interior of the stones, cross sections of laminated matter was present, and in the case of compound uroliths, a pure silica nucleus with stones composed of mixtures of silica with calcium oxalate or struvite were observed.

By measuring the hardness of different types of uroliths, we observed that silicate uroliths break at an average pressure of 36.79 kgf; above the uroliths of other types of minerals such as struvite, calcium oxalate, cystine, and ammonium urate that break at a pressure between 6.67–12.94 kgf.

### 3.6. Urinary pH

The urinary pH of the dogs with silica uroliths at the time of diagnosis ranged from 5 to 9, with a median of 6.5.

## 4. Discussion

The formation of silica uroliths in the urinary tract is related to urinary hyperexcretion of silica, secondary to a constant consumption of high concentrations of an absorbable form of silica obtained from the solid diet or dissolved in the water [26].

In the case of dogs, it is very likely that pet foods of animal origin do not contain a high content of silicates [27]. There is only one hypothesis for the development of the pathophysiology, which is the consumption of low-quality pet food for dogs, produced using vegetable ingredients such as corn gluten, rice husk, soybean husk, wheat husk, and beet pulp as an added source of protein or soluble fiber, which can have a high content of silicates, as in plants, silica is absorbed by the roots and deposited in the cell walls as silica, soluble silicates, and other organic combinations with the highest concentration in leaves and stems [16]. However, it is important to mention that a higher content of silicates in the solid diet does not specifically increase their intestinal absorption and urinary excretion, as some particles of silicon in the diet have a very low intestinal absorption [26]. Another possible cause is the increased consumption of silica in the water, as the particles are highly soluble, presenting high gastrointestinal absorption and rapid excretion in the urine [28]. Therefore, the chronic consumption of water from aquifers located in volcanic areas is considered a possible risk factor [29].

In the analysis of the epidemiology of urolithiasis in Mexico, we detected a frequency of 12.9% of pure uroliths among the different cases of silica urolithiasis. Other high frequencies have been documented only in the United States 6.7%, Switzerland 8% [6, 9], and in localized geographic areas in Mexico 9.2 and 13.3% [4, 21]. It was also possible to detect in mixed uroliths and compound uroliths different proportions of silica with mixtures of other minerals such as struvite, calcium oxalate, cystine, and ammonium urate, increasing the frequency of silica-containing uroliths to 17.4% of the population studied. Other authors have also mentioned a mixture of silica with other minerals such as struvite and calcium oxalate stones [18].

The higher frequency of silica urolithiasis in Mexico compared to the other parts of the world can be associated with the geographical location of these animals as 98.9% of the cases were located in the central region of the country and only 1.1% was located in the northern region. This indicates a geographical risk factor, as the cases occurred in populations that are located in the central region of the country, immersed within the Trans Mexican Neovolcanic axis, which is a strip of volcanoes that extends transversely from the Pacific Ocean to the Gulf of Mexico [30]. This is similar to a report from Switzerland, which found a higher proportion of cases of silica urolithiasis in the eastern parts of the country than in the western parts, which might be associated with a geographical factor [6]. In the case of the studied population, it is likely that the water consumed by these dogs contains a high concentration of silica as it comes from groundwater that is near the volcanoes, which has been observed as a possible risk factor for the formation of the silica urolithiasis. However, future studies on the concentration of silica in the water from different regions of the country are needed to verify this hypothesis.

Analysis of the epidemiological data of the population of dogs revealed that urolithiasis presented from 5 months to 14 years. Thus, urolithiasis is not only prevalent in adult dogs but also in young ones, as in the case of a puppy aged 5 months, which corroborates data reported by Osborne et al. [16]. However, dogs are most frequently affected after 6 years of age, demonstrating a significant risk from this age onward. A significantly higher presentation was observed in males, and other studies have also noted this [9, 16, 18]. In fact, in a previous study by the group of Del-Angel-Caraza et al. [4] in Mexico, the condition was only observed in males. This higher frequency in males might be related to the anatomy of the urethra, which is narrow and long in males, causing the uroliths to be retained, resulting in a urethral obstruction in some cases, whereas the female urethra is shorter and more lax, allowing small uroliths to be expelled with less difficulty during urination before clinical signs appear.

Silica urolithiasis can occur in different breeds. In this study, 33 different breeds were analyzed, and the six most representative breeds were the Labrador, Miniature Schnauzer, Crossbreed, German Shepherd, Golden retriever, and Poodle, which is very similar to data in other reports [6, 16, 21]. Our study demonstrated a significant risk to the Labradors and Golden Retrievers, although other studies have found that the German Shepherd is more at risk [18, 19]; however, in our study there was no evidence of predisposition. It should be noted that the crossbreed dogs do not belong to any particular breed; however, they were within the group of the most frequently affected dogs, which has also been reported in other studies [9, 18]. Of the dogs that could be classified by breed size, the large breeds (79.83%) were the most representative and had a significant risk in agreement with other studies [4, 6, 21]. One possible explanation is that large breed dogs consume larger volumes of water and produce a higher urinary volume [31], which might favor a higher concentration of this mineral in the urine. However, pure silica uroliths can also occur in small breeds, although less frequently; or find small amounts of silicate in mixed uroliths [9].

The uroliths were detected mainly in the lower urinary tract: 98.25% were in the bladder and urethra, which has also been described in other populations [18, 32], and just 1.14% was found in the ureter and kidney; it is possible to find them in different anatomical sites simultaneously. This is unlike the Kenya study, where the uroliths were found in the kidney [17].

Pure silica uroliths can have a spherical shape with radial projections of different length, thickness, and number (Jackstone). These structures favor the trapping of these uroliths in the urethra in males by adhering to the urethral mucosa and causing an obstruction. Uroliths can be present as singles or multiples, have different colors and sizes measuring up to 35 mm, and tend not to be very heavy, despite having a maximum weight of 22 g. However, the uroliths containing silica with mixtures of other compounds in different proportions (struvite, calcium oxalate, cystine) adopted different forms; they were circular, square, irregular, or oval, and were larger and heavier. This is consistent with what was described by Osborne [16], as the characteristics of a urolith depend on its age, the site of the growth, and especially the purity of the chemical components found in the uroliths. Based on the analysis of the hardness carried out on samples of uroliths with the same shape, size, and weight, differing only in their mineral composition, we note that the silica urolith was the hardest, requiring a force of 36.79 kgf to break it, whereas the other compounds broke with a lower force of 12.94–6.67 kgf. One possible explanation is that silica uroliths have laminated cross sections in their interior, which might make them very strong and prevent rupture; however, these observations were limited to the analysis of only a single sample per type of mineral; hence, it is necessary to carry out more studies in the future.

When analyzing the results of the urinalysis of the dogs, the pH of the urine at the time of diagnosis ranged from acid to alkaline, with a range from 5 to 9, and a median of 6.5. It has been reported that the urinary pH does not influence the precipitation of silica stones; however, it appears that they tend to form in a neutral to acidic pH, and it is probable that when the clinical picture presents with a urinary tract infection, an alkaline pH is found [32].

## 5. Conclusion

In the population studied, the risk factors for silica urolithiasis were gender, with males being the most affected; age, with a higher frequency in dogs over 6 years of age; large breed dogs, such as Labradors and Golden retrievers; and being located in the regions within the Trans Mexican Neovolcanic axis located in the central axis of the country. The possibility that geographical location is a risk factor for silica urolithiasis should be confirmed in future studies.

## Figures and Tables

**Table 1:** Mineral composition of uroliths from dogs between 2005 and 2018.

Type of mineral	Number	%
Struvite	611	44.17
Calcium oxalate	372	26.89
Silica	179	12.94
Purines	57	4.12
Cystine	17	1.22
Calcium phosphate	13	0.93
Mixed uroliths	105^*∗*^	7.59
Compound uroliths	29^*∗∗*^	2.09

Total	1383	100

^*∗*^42.8% of these uroliths contained 40% silica mixed with other compounds in different quantities. ^*∗∗*^55.1% of these uroliths had a pure nucleus of silica.

**Table 2:** Risk by age group.

Age	Silica urolithiasis, *n* (%)	OR	95% CI	*p*
<5 years	79 (44)	0.679	0.494–0.933	0.016
6–10 years	100 (55.9)	1.690	1.189–2.402	0.003

**Table 3:** Distribution of frequency of silica urolithiasis (SiU) by breed and gender of dogs, and the risk for the breed compared to other breeds without urolithiasis or with another type of urolithiasis.

Breed	SiU (*n*)	Gender		OR	95% CI	*p*
		Male	Female			
Labrador	32	32	0	3.00	1.664–5.423	<0.001
Schnauzer	21	18	3	1.21	0.687–2.160	0.499
Crossbreed	17	16	1	1.69	0.918–3.142	0.092
German shepherd	16	16	0	1.60	0.791–3.252	0.190
Golden retriever	16	16	0	2.78	1.235–6.278	0.014
Poodle	12	12	0	1.39	0.701–2.794	0.341

## Data Availability

The data used to support the findings of this study are available from the corresponding author upon request.
